# Increased PAI-1 plasma levels and risk of death from dengue: no association with the 4G/5G promoter polymorphism

**DOI:** 10.1186/1477-9560-3-17

**Published:** 2005-11-07

**Authors:** ATA Mairuhu, TE Setiati, P Koraka, CE Hack, A Leyte, SMH Faradz, H ten Cate, DPM Brandjes, ADME Osterhaus, PH Reitsma, ECM van Gorp

**Affiliations:** 1 Department of Internal Medicine, Slotervaart Hospital, Louwesweg 6, 1066 EC Amsterdam, The Netherlands; 2 Department of Paediatrics, Dr. Kariadi Hospital, Semarang, Indonesia; 3 Institute of Virology, Erasmus Medical Centre, P.O. Box 1738, 3000 DR Rotterdam, The Netherlands; 4 Department of Immunopathology, Sanquin Research, P.O. Box 9190, 1006 AD Amsterdam, The Netherlands; 5 Laboratory for Experimental and Clinical Immunology, Academic Medical Centre, Meibergdreef 9, 1100 DD Amsterdam, The Netherlands; 6 Department of Clinical Chemistry, VU Medical Centre, Amsterdam, The Netherlands; 7 Hematology and Clinical Chemistry Laboratory, Onze Lieve Vrouwe Gasthuis, Amsterdam, The Netherlands; 8 Molecular and Cytogenetics Unit, Biotechnology Laboratory, Medical Faculty Diponegoro University, Semarang, Indonesia; 9 Department of Internal Medicine, University Hospital Maastricht, P.O. Box 5800, 6202 AZ Maastricht, The Netherlands; 10 Cardiovascular Research Institute, Maastricht University, P.O. Box 616, 6200 MD Maastricht, The Netherlands; 11 Laboratory for Experimental Medicine, Academic Medical Centre, Meibergdreef 9, 1100 DD, Amsterdam, The Netherlands

## Abstract

**Background:**

Dengue virus infected patients have high plasminogen activator inhibitor type I (PAI-1) plasma concentrations. Whether the insertion/deletion (4G/5G) polymorphism in the promotor region of the PAI-1 gene is associated with increased PAI-1 plasma concentrations and with death from dengue is unknown. We, therefore, investigated the relationship between the 4G/5G polymorphism and PAI-1 plasma concentrations in dengue patients and risk of death from dengue.

**Methods:**

A total of 194 patients admitted to the Dr. Kariadi Hospital in Semarang, Indonesia, with clinical suspected severe dengue virus infection were enrolled. Blood samples were obtained on day of admission, days 1, 2 and 7 after admission and at a 1-month follow-up visit. Plasma concentrations of PAI-1 were measured using a sandwich ELISA kit. The PAI-1 4G/5G polymorphism was typed by allele-specific PCR analysis.

**Results:**

Concentrations of PAI-1 on admission and peak values of PAI-1 during admission were higher than the values measured in healthy controls. Survival was significantly worse in patients with PAI-1 concentrations in the highest tertile (at admission: OR 4.7 [95% CI 0.9–23.8], peak value during admission: OR 6.3 [95%CI 1.3–30.8]). No association was found between the PAI-1 4G/5G polymorphism, and PAI-1 plasma concentrations, dengue disease severity and mortality from dengue.

**Conclusion:**

These data suggest that the 4G/5G polymorphism has no significant influence on PAI-1 concentrations in dengue virus infected patients and is not associated with the risk of death from dengue. Other factors contributing to the variability of PAI-1 plasma concentrations in patients with dengue need to be explored.

## Background

Dengue is the most prevalent viral disease transmitted by arthropod vectors worldwide [1]. An estimated 50–100 million cases of dengue fever and 500.000 cases of dengue haemorrhagic fever resulting in around 24.000 deaths occur annually depending on epidemic activity [1,2]. At present, almost 30% of the world population is at risk for dengue virus infection and it is expected that this number will increase substantially as transmission spreads to other yet unaffected geographic regions [3]. The viruses are transmitted to humans through infected mosquitoes, and may induce clinical manifestations ranging from a mild, uncomplicated febrile illness to the more severe dengue haemorrhagic fever and dengue shock syndrome. Increased vascular permeability is thought to be central in the pathogenesis of dengue haemorrhagic fever and dengue shock syndrome, since it results in the loss of plasma from the vascular compartment, which may give rise to shock in severe cases. Bleeding manifestations are believed to result from thrombocytopenia and thrombocytopathia, but increasing evidence suggests an important role for other abnormalities in the coagulation and fibrinolytic systems [4].

Nearly all patients suffering from dengue haemorrhagic fever show some evidence of a deranged coagulation system, but coagulation abnormalities are most marked in dengue shock syndrome patients [5]. The activity of the fibrinolytic system is amongst others regulated by plasminogen activator inhibitor type I (PAI-1), of which levels may greatly increase during acute phase reactions. Indeed, levels of PAI-1 are high in particular in dengue shock syndrome patients with an adverse clinical outcome [6,7]. A single base pair insertion/deletion (4G/5G) polymorphism in the promotor region of the PAI-1 gene has been associated with increased plasma concentrations of PAI-1 and with the development of shock and death after infection with Neisseria meningitides [8,9]. We therefore investigated whether in dengue virus infected patients increased PAI-1 levels are associated with a greater risk of death from dengue and whether the 4G/4G genotype contributes to these higher levels.

## Methods

Patients were selected from two observational studies conducted in the Dr. Kariadi Hospital in Semarang, Indonesia. In the first study, performed from 1996 to 1997 at the paediatric intensive care unit, a total of 50 patients with a clinical diagnosis of suspected dengue shock syndrome were studied. Blood samples were obtained from these patients on day of admission and on days 1, 2 and 7 after admission. The study protocol and the results of the measurement of PAI-1 plasma levels have been described previously [6]. The second study was performed from 2001 to 2003 at the paediatric intensive care unit and the paediatric ward. Patients, aged 3 to 14 years, admitted to the hospital with a clinical diagnosis of suspected dengue shock syndrome or with a clinical diagnosis of suspected dengue haemorrhagic fever were included. Demographic data, medical history, physical examination findings and subsequent progress for each patient were recorded on a standard data form. Blood samples were obtained on day of admission, days 1, 2 and 7 after admission and at a 1-month follow-up visit. A formal classification, according to WHO criteria [2], using all available clinical and laboratory data, was done after completion of both studies. If dengue virus infected patients did not meet the criteria for dengue haemorrhagic fever or dengue shock syndrome, they were considered to suffer from dengue fever. The controls were healthy school-aged children who had no history of dengue haemorrhagic fever or dengue shock syndrome and originated from the same geographical area as the cases.

All blood samples were centrifuged within 1–2 hours after retrieval at 15°C for 20 minutes at 1600*g. Plasma was separated, stored at -80°C and assayed batch-wise in the Netherlands after transportation on dry ice. Since coagulation test results are affected by poor sample quality, only test results of samples with no visible haemolysis or clot formation were included in this analysis. Plasma concentrations of PAI-1 were measured using two separate assays. A sandwich ELISA kit that has been described by de Boer et al [10], was used in the 1996–1997 study. In the second study a commercially available sandwich ELISA kit (Imulyse, Biopool, Sweden) was used. Genomic DNA from patients was prepared either from EDTA whole blood by means of a salting out method as described elsewhere or in case only plasma samples were available with use of the Invisorb^® ^Spin Cell Mini Kit (Invitek GmbH, Berlin, Germany) [11]. The insertion/deletion (4G/5G) polymorphism in the promotor region of the PAI-1 gene was typed by allele-specific PCR and RFLP analysis [11]. The ethics committee of the Dr. Kariadi Hospital approved all clinical and laboratory aspects of both studies. Blood samples were taken from patients and controls provided that a parent or legal guardian gave informed consent.

Paired blood samples from both studies were tested for serologic evidence of acute dengue infection. A commercially available capture and indirect ELISA (Focus Technologies, Cypress, Calif., USA) was used for the detection of dengue virus specific IgM and IgG antibodies respectively. This was performed according to the procedures described by the manufacturer [12]. For some patients, a definitive serodiagnosis was not possible because no convalescent sample was obtained. Detection of dengue antigen and RNA was attempted in these cases using a dot blot immunoassay and a dengue serotype specific revere transcriptase PCR respectively [13]. Patients with serologic evidence of acute dengue infection, a positive dot blot and/or positive PCR were considered to have confirmed dengue virus infection. Those with definite negative serology and/or a well-substantiated alternative clinical diagnosis were classified as not dengue. In the absence of a well-substantiated alternative clinical diagnosis and with inconclusive serology patients were classified as indeterminate.

Categorical data are expressed as numbers and frequencies, and were compared by means of χ^2 ^analysis. Continuous data are expressed as medians with corresponding interquartile ranges and were compared by means of the Kruskal Wallis test. Since PAI-1 plasma levels were measured using two different assays, the nonparametric regression procedure of Passing and Bablok was used to compare these two methods and to validate recalculation of one of the datasets to pool the data [14]. For this purpose, 39 samples were analysed with both assays. The Passing and Bablok regression equation resulting from this comparison is given in the *Results *section, together with the 95% confidence intervals for the estimates of slope and intercept. PAI-1 plasma levels obtained from the 1996–1997 cohort were converted using the regression equation. We subsequently divided PAI-1 plasma levels on admission and peak PAI-1 plasma levels during admission into tertiles of similar size. Odds ratios and the corresponding 95% confidence intervals (95% CI) were estimated by cross-tabulation using the lowest tertile as reference category. To determine the association between PAI-1 concentration and mortality independent of age, sex, year project and plasminogen activator inhhibitor-1 polymorphism, we used logistic regression analyses. A P-value ≤ 0.05 was considered to indicate statistical significance. Analyses were performed using SPSS 11.0.1. Method comparison was performed using Analyse-it Clinical Laboratory statistics module version 1.62 for Microsoft Excel.

## Results

Of 233 enrolled patients with suspected dengue haemorrhagic fever or dengue shock syndrome admitted to the paediatric intensive care unit and the paediatric ward of the Dr. Kariadi Hospital, 202 (87%) were confirmed to have acute dengue, 3 (1%) were categorised as definitely not dengue and 28 (12%) as indeterminate. When a formal classification using the criteria set by the WHO was performed, 106 (52%) were classified as having dengue shock syndrome, 76 (38%) as having dengue haemorrhagic fever and 20 (10%) as having dengue fever. Nineteen of 202 patients with confirmed dengue (9%) died during follow up.

Genomic DNA was obtained from 194 patients. The remaining patients could not be typed because of insufficient volume of blood left or low yield of DNA. Clinical features and basic laboratory investigations on admission of the 194 patients are summarised in Table 1. The numbers of 4G/4G, 4G/5G and 5G/5G PAI-1 genotypes among patients in relation to mortality are summarised in Table 2. Of 192 control samples tested, 45 patients (23%) were 4G/4G homozygous, 83 (43%) were 4G/5G heterozygous, and 64 (33%) were 5G/5G homozygous. The frequencies of the PAI-1 promoter genotypes 4G/4G, 4G/5G, and 5G/5G did not differ significantly between the 1996–1997 project, the 2001–2003 project and the control group (P = 0.520). The proportion of deaths among patients with the 4G/4G, 4G/5G and 5G/5G genotype did not differ significantly (1996–1997 project: P = 0.979; 2001–2003 project: P = 0.986; two projects combined: P = 0.781). The genotype frequencies among patients with respect to final clinical diagnosis according to the criteria set by the WHO are summarised in Table 3. Results indicate that the PAI-1 promoter genotypes are not associated with dengue disease severity (P = 0.508).

**Table 1:** Clinical characteristics and laboratory findings

Characteristic	
Age, median (IQR), years	6 (4–10)
Male sex, n (%)	92 (47)
Duration of symptoms, median (range), days	4 (1–7)
Systolic blood pressure, median (IQR), mmHg	90 (80–100)
Pulse pressure <20 mmHg, n (%)	22 (11)
Spontaneous bleeding, n (%)	118 (62)
Haematocrit, median (IQR), %	41 (36–45)
Platelet count, median (IQR), cells *10^3^/mm^3^	58 (37–85)

° IQR denotes 25th and 75th interquartile range, n denotes number of patients.

**Table 2:** Clinical outcome of dengue virus infected patients classified by PAI-1 genotype

	1996–1997			2001–2003		
Genotype	All patients	Survivors	Non-survivors	All patients	Survivors	Non-survivors

G4/G4	8 (18%)	6 (14%)	2 (5%)	33 (22%)	32 (21%)	1 (1%)
G4/G5	15 (34%)	11 (25%)	4 (9%)	62 (41%)	60 (40%)	2 (1%)
G5/G5	21 (48%)	15 (34%)	6 (14%)	55 (37%)	53 (35%)	2 (1%)
Total	44 (100%)	32 (73%)	12 (27%)	150 (100%)	145 (97%)	5 (3%)

**Table 3:** Dengue disease severity and PAI-1 genotype

Genotype	DF	DHF	DSS
G4/G4	6 (3%)	13 (7%)	22 (11%)
G4/G5	6 (3%)	35 (18%)	36 (19%)
G5/G5	8 (4%)	27 (14%)	41 (21%)
Total	20 (10%)	75 (39%)	99 (51%)

**Figure 1:**
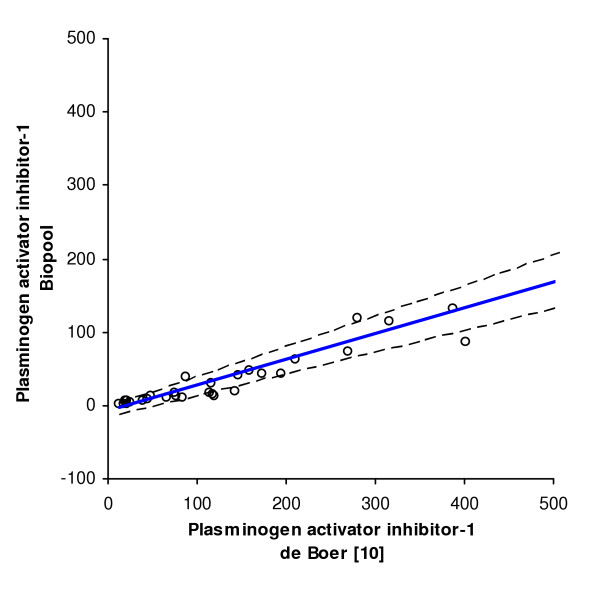
**Comparison of two PAI-1 assays**. Comparison of PAI-1 plasma levels obtained by a Biopool assay and an assay previously described by de Boer and colleagues [10]). The Passing & Bablok regression equation is: y = 0,3505x - 7,4227 ng/ml; n = 39. *Solid line*, regression line; *dashed lines*, 95% CI for the regression line.

**Figure 2:**
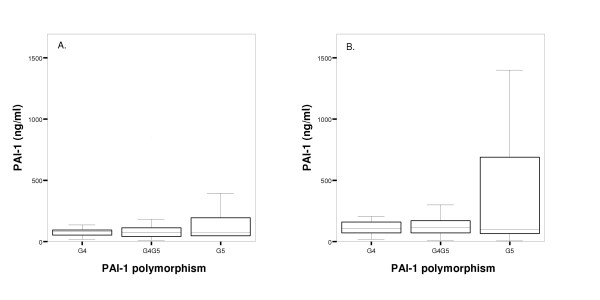
**Relation between PAI-1 plasma concentrations and PAI-1 genotype in dengue virus infected patients**. Box-and-whisker plots of PAI-1 plasma concentrations on admission (A.) and peak values during admission (B.). The central line in the box plot represents the median, the boxed areas represent the interquartile ranges. Whiskers at the ends of the box show the distance from the end of the box to the largest and smallest observed values that are less than 1.5 box lengths from either end of the box.

PAI-1 plasma levels were measured in 124 patients from whom sufficient frozen plasma samples was available with the use of two separate ELISA's. Thirty-nine random plasma samples were used for the comparison of the two assays. PAI-1 level measurements are known to be dependent on the assay employed, since not all antibodies used have the same specificity with respect to the various molecular forms of PAI-1 in blood (active PAI-1, PAI-1 complexed with vitronectin, inactive or latent PAI-1 and PAI-1 complexed with its target proteases tissue-type and urokinase-type plasminogen activator) [15]. Passing & Bablok regression analysis (Figure 1), however, clearly showed that the two assays employed in this study have a straightforward linear relationship between their outcomes. This allowed us to convert the PAI-1 levels measured in the 1996–1997 cohort to levels that would have been obtained with the assay used in the second cohort, using the Passing and Bablok regression equation (an intercept of -7.42 ng/ml (95% CI, -16.39 to -3.24 ng/ml) and slope of 0.35 (95% CI, 0.30 to 0.42)). Concentrations of PAI-1 on admission (71 ng/ml [42–118]) and peak values during admission (96 ng/ml [64–199]) were higher than the concentrations measured in the healthy control group (30–60 ng/ml). Patients included in the 2001–2003 project and diagnosed with DSS had higher PAI-1 plasma levels on admission (P = 0.002) and during admission (P < 0.001) than those diagnosed with DF or DHF (Table 4). However, no statistical significant difference was present between patients with different dengue disease severities when both projects were combined (PAI-1 plasma levels on admission: P = 0.212 and during admission: P = 0.089). Survival was significantly worse in patients with PAI-1 concentrations in the highest tertile (Table 5). As shown by multiple logistic regression analysis, the odds ratio for the risk of death – adjusted for age, sex, year project and plasminogen activator inhibitor-1 genotype – of PAI-1 levels at admission in the highest tertile was 7.57 (95%CI, 1.16–49.30). The adjusted odds ratio for the risk of death of peak PAI-1 levels during admission in the highest tertile was 7.41 (95%CI, 1.30–42.26). Figure 2 illustrates the relation between PAI-1 plasma concentrations and the PAI-1 genotypes. Differences were not statistically significant (values at admission: P = 0.919; peak values during admission: P = 0.470).

**Table 4:** Relation between PAI-1 plasma levels and disease severity°

Project	PAI-1	DF	DHF	DSS
	(ng/ml)	Median (IQR)	Median (IQR)	Median (IQR)
1996–1997¶	Admission	-	-	56.7 (33.0–130.2)
	Peak values	-	-	91.6 (37.7–343.6)
2001–2003	Admission	77.0 (69.0–99.0)	60.0 (42.3–82.5)	107.0 (52.0–670.0)
	Peak values	82.0 (69.0–129.0)	78 (59.3–118.3)	264.0 (117.0–844.0)
Total	Admission	77.0 (69.0–99.0)	60.0 (42.3–82.5)	87.0 (35.0–180.8)
	Peak values	82.0 (69.0–129.0)	78 (59.3–118.3)	130.7 (53.7–531.1)

° IQR denotes 25th and 75th interquartile range.

¶ Only DSS patients were included in the 1996 project.

**Table 5:** Mortality risk for plasminogen activator-1 plasma levels.*

PAI-1	ng/ml	Number of patients	Number of deaths	OR (95% CI)	OR_adj _(95% CI)°
Value at admission	<50	41	2	1	1
	50–94	40	3	1.6 (0.3–10.0)	3.3 (0.4–25.0)
	>94	41	8	4.7 (0.9–23.8)	7.6 (1.2–49.3)
Maximum value during admission	<72	41	2	1	1
	72–151	42	3	1.5 (0.2–9.5)	2.1 (0.2–18.2)
	>151	41	10	6.3 (1.3–30.8)	7.4 (1.3–42.3)

* CI, denotes confidence interval; OR, denotes odds ratio; OR_adj_, denotes adjusted odds ratio

° Risk of death adjusted for age, sex, year project and plasminogen activator inhhibitor-1 polymorphism. Lowest tertile was used as reference category.

## Discussion

This study was undertaken to investigate the relationship between PAI-1 plasma concentrations and clinical outcome of dengue virus infections, and to establish whether PAI-1 plasma concentrations in dengue virus infected individuals are associated with the 4G/5G promotor polymorphism in the PAI-1 gene. We and others have previously found increased PAI-1 plasma concentrations in patients with severe dengue in particular in those with a poor clinical outcome [5,7]. Since a genetic predisposition to produce high PAI-1 plasma concentrations appears to be associated with poor clinical outcome in Neisseria meningitides infections [8,9], we hypothesised that dengue virus infected individuals carrying the 4G/4G genotype have higher PAI-1 plasma concentrations and are therefore at increased risk of death. Consistent with previous studies, we found increased PAI-1 concentrations in dengue virus infected individuals. However, PAI-1 plasma concentrations were not related to dengue disease severity, but were significantly associated with death from dengue. No significant association between PAI-1 plasma concentrations and carriage of the 4G/4G genotype was observed. The frequencies of the three genotypes between survivors and non-survivors, and between patients with different disease severities were not different. These findings suggest that increased PAI-1 plasma concentrations, and dengue disease severity and mortality are not dependent on the 4G polymorphism in the PAI-1 gene in this population.

An increased risk of death in dengue virus infected patients with high PAI-1 plasma concentrations adds to findings of PAI-1 levels being able to predict lethality in patients with bacterial sepsis in a very sensitive way [16-20]. One of the primary roles of PAI-1 *in vivo *is to inhibit tissue-type plasminogen activator, the major proteolytic activator of plasminogen [21,22]. By inhibiting fibrinolytic activity, increased PAI-1 concentrations may contribute to a procoagulant state leading to an increased deposition of fibrin and formation of microthrombi with subsequent multiorgan failure and death. A variety of cells, including endothelial cells, hepatocytes and platelets, synthesize and secrete PAI-1 in response to inflammatory stimuli such as interleukin-1 and tumour necrosis factor [10,22-24]. The release of these inflammatory mediators by monocytes and T lymphocytes activated by dengue virus may well contribute to the over-production of this inhibitor of fibrinolysis [25-27]. Dawson and colleagues showed that the common insertion/deletion (4G/5G) polymorphism in the promotor region of the PAI-1 gene affects the response of the gene to acute phase stimuli [23]. The 4G allele produced six times more mRNA than the 5G allele in response to interleukin-1 [23]. Eriksson and colleagues, however, were unable to reproduce these findings and based on their study results they concluded that the insertion/deletion (4G/5G) polymorphism is not related to an allele-specific response to interleukin-1 [28]. Instead, they found that the insertion/deletion (4G/5G) polymorphism influences basal PAI-1 transcription only [28].

Apparently other underlying mechanisms not related to the 4G/5G polymorphism must be involved in the increase in PAI-1 levels found in dengue virus infected individuals. This might include clearance impairment rather than or in addition to stimulation of synthesis. It is interesting to note that PAI-1 is cleared from the circulation by the liver [29]. Indeed in patients with severe liver disease, PAI-1 has been shown to be increased as a result of a decrease in hepatic clearance [30,31]. Since hepatic dysfunction is a relatively common finding in severe dengue virus infections, it is possible that a less efficient clearance contributes to increased PAI-1 levels in dengue virus infected individuals [32-34]. Previous studies investigated factors that could potentially influence PAI-1 levels, including environmental factors, metabolic determinants, ethnicity and a variety of other polymorphisms within the PAI-1 gene [35-38]. It remains unclear whether and to what extent these factors contribute to the variability in PAI-1 levels in dengue virus infected individuals. Previously studied individuals were either healthy, were patients with coronary artery disease, or were patients with diabetes mellitus. Clearly these study populations cannot be compared to patients suffering from a severe infectious disease that is characterised by an overwhelming inflammatory response.

Several potential limitations of the present study should be noted. The 1996–1997 cohort was characterized with a high mortality rate of 27%. Although the exact reason for this high mortality remains to be determined, it is likely that it results from a combination of factors. Our study was performed in a Tertiary Hospital that serves a large part of Middle-Java. Patients may travel long distances to be treated in this hospital and it could well be that they are presented late in the course of disease. Initial fluid resuscitation according to WHO guidelines is generally insufficient in these cases and patients usually end up in profound shock. Despite admission at the Pediatric Intensive Care Unit mortality rate is high. In addition, the 1996–1997 rainy season was characterised by high numbers of patients admitted to hospitals because of DHF/DSS and high number of non-survivors. It is believed that an unusually virulent virus circulated that year although microbiological sampling could not be performed at that time because of limited resources.

Study size is an important issue in the establishment of an association between the insertion/deletion (4G/5G) polymorphism in the PAI-1 gene and clinical outcome. Hermans and colleagues previously found an association between the homozygous 4G deletion polymorphism and mortality from Neisseria meningitides infection among 129 patients from two different cohort groups [8]. This association was also observed when the largest of the two cohort groups was studied separately, but was not seen in the smallest group in which only 37 patients were included. In order to obtain a sufficient number of patients, we therefore decided to combine the results of two different projects. This decision was based on the fact that these two projects included patients with the same ethnic background, used similar trial procedures and applied uniform diagnostic and clinical management procedures. Although mortality rates between the 1996–1997 cohort and the 2001–2003 cohort differed considerably, one must realise that in the 1996–1997 cohort only DSS patients were included. In the 2001–2003 cohort also included patients who had no evidence of circulatory failure were included. Mortality rate among DSS patients included in the 2001–2003 cohort was 18%. Our findings of similar frequencies of the PAI-1 genotypes within the 1996–1997 project and the 2001–2003 project supports our decision to combine both groups.

In conclusion, this study demonstrates that high PAI-1 plasma levels are associated with an increased risk of death from dengue without the 4G/5G polymorphism in the promotor of the gene for PAI-1 playing a role. Additional studies are needed to explore the possibility of other polymorphisms within the PAI-1 gene and factors, like ethnicity or environmental factors, contributing to the variability of PAI-1 plasma concentrations in patients with dengue.

## Competing interests

The author(s) declare that they have no competing interests.

## Authors' contributions

A.M., T.S. and P.K. wrote the first draft of the study protocol. H.C., D.B., A.O. and E.G. contributed to the writing of the study protocol. A.M. and T.S. were responsible for implementation of the study. T.S. was responsible for management of patients, and data collection at the study site. C.H., A.L., S.F. and P.R. were responsible for the measurement of PAI-1 plasma levels and for typing of the polymorphism. P.K. and A.O. were responsible for all diagnostic procedures. A.M. and A.L. did all statistical analyses. A.M., A.L., H.C., and E.G. wrote the first draft of the report, and all other authors contributed at subsequent stages.
